# UBC9 coordinates inflammation affecting development of bladder cancer

**DOI:** 10.1038/s41598-020-77623-9

**Published:** 2020-11-26

**Authors:** Xiaoliang Huang, Yuting Tao, Jiamin Gao, Xianguo Zhou, Shaomei Tang, Caiwang Deng, Zhiyong Lai, Xinggu Lin, Qiuyan Wang, Tianyu Li

**Affiliations:** 1 Department of Urology and Nephrology, The First Affiliated Hospital of Guangxi Medical University, Nanning, China; 2 Center for Genomic and Personalized Medicine, Guangxi Medical University, Nanning, Guangxi Zhuang Autonomous Region China; 3 Guangxi Key Laboratory for Genomic and Personalized Medicine, Guangxi, Collaborative Innovation Center for Genomic and Personalized Medicine, Nanning, Guangxi Zhuang Autonomous Region China; 4 Key Laboratory of Longevity and Ageing-Related Disease of Chinese Ministry of Education, Center for Translational Medicine and School of Preclinical Medicine, Guangxi Medical University, Nanning, Guangxi Zhuang Autonomous Region China

**Keywords:** Bladder cancer, Cancer epigenetics, Urological cancer

## Abstract

Dysregulation of SUMO modification is linked to carcinogenesis. UBC9 is the sole conjugating enzyme in sumoylation and plays a pivotal role in maintaining homeostasis and restraining stress reactions. However, the clinical significance and function of UBC9 in bladder cancer remain unclear. In this study, immunohistochemistry was used to determine the expression of UBC9. UBC9 knock-down and SUMO inhibition were conducted followed by proliferation, migration, and cell cycle assays. RNA sequencing and bioinformatic analysis were used to identify potential mechanisms of UBC9. Cytokine membrane antibody array was used to detect the expression of cytokine. The mass cytometry TOF (CyTOF) was used to explore the association between bladder cancer stem cell-like population and UBC9 expression. Our results showed that UBC9 played a dual role in bladder cancer. UBC9 was up-regulated in bladder cancer, but was negatively correlated with TNM stage and grade. Knocking-down of UBC9 resulted in dramatic activation of inflammatory gene expression, which might cause inhibition of cell proliferation and inducing cell apoptosis. IL6 was the hub gene in UBC9 regulatory network. Markedly up-regulated IL6 after knocking-down of UBC9 activated the expression of CD44, which was a prominent marker of cancer stem cells. Thus, our results revealed an important and previously undescribed role for UBC9 in modulation of inflammatory signaling of bladder cancer. UBC9 in bladder cancer cells is required to maintain high sumoylation levels and alleviate stress-related inflammation threats to cell survival. Lacking UBC9 contributes to inflammation activation, epithelial–mesenchymal transition and stem cell-like population formation, leading to cancer progression.

## Introduction

Bladder cancer is the fourth most common cancer in men and the ninth most common worldwide^1^. Although many treatment options, such as radical cystectomy, transurethral resection of bladder tumor (TURBT), systemic chemotherapy are available, the prognosis of invasive bladder cancers still remains poor^2^. Despite the important progressive of targeted therapy in several cancers, there is still no satisfactory targeted therapeutic reagent for bladder cancer^3^. Thus, it is urgently needed to discover the associated molecular mechanisms in the tumorigenesis and development of bladder cancer and identify potential biomarkers to improve the diagnosis and treatment of bladder cancer.

Ubiquitin-conjugating enzyme 9 (UBC9) is the sole conjugating enzyme for an essential posttranslational protein modification called sumoylation. This process resembles the ubiquitin pathway in terms of protein maturation, activation, conjugation, and ligation^4^. UBC9 transfers activated small ubiquitin-related modifier (SUMO) to various protein substrates. At least 1,000 human proteins have been confirmed to be connected with SUMO^5^. Importantly, recent advances have clearly demonstrated that sumoylation is critical for homeostasis and modulation of cellular response under stress^6,7^. Malignant cells face stress more frequently because their mechanisms of DNA damage sensing and repair are compromised. Furthermore, the tumor microenvironment typically experiences hypoxia and oxidative stress that stimulates cytokines and triggers inflammation^8^. Many cancer cells therefore enhance sumoylation to mitigate excessive inflammatory responses under intrinsic and extrinsic (e.g., chemotherapy drugs) stresses^9^.

The regulation of UBC9 is a reflection of sumoylation degree^10^. Thus, UBC9 has emerged as a potential target for cancer therapy^11^. Knocking out UBC9 enhances sensitivity to anticancer drugs^12,13^, while small-molecule UBC9 inhibitors, such as 2-D08 and spectomycin B1, show some promise in ameliorating cancers in vitro and ex vivo^14,15^. However, the clinical significance and regulatory mechanism of UBC9 in bladder cancer remain poorly understood. Here, we reported a dual role of UBC9 in bladder cancer. UBC9 in bladder cancer cells is required to maintain high sumoylation levels and alleviate stress-related inflammation threats to cell survival, while lack of UBC9 contributes to stem cell-like population formation and cancer progression by activated inflammation.

## Materials and methods

### Clinical specimens

In the present study, 158 cases of bladder cancer samples and 14 cases of adjacent tissues were obtained from patients at the First Affiliated Hospital of Guangxi Medical University between 2015 and 2019. Among all the samples, 106 cases of bladder cancer samples and 14 cases of adjacent tissues were used for immunohistochemistry. The remaining 52 samples were subjected to RNA sequencing. All of the patients were primary bladder cancer without chemotherapy or radiotherapy before the collection of the tissues. Written informed consents were obtained from all patients. The study was approved by the Ethics and Human Subject Committee of Guangxi Medical University. All experiments and methods were performed according to relevant guidelines and regulations formulated by the Guangxi Medical University.

### Public datasets download

The level three RNA sequencing (RNA-seq) data of The Cancer Genome Atlas (TCGA) Urothelial Bladder Carcinoma and corresponding clinical information were downloaded from The Genomic Data Commons (GDC) Data Portal (https://portal.gdc.cancer.gov/). In addition, three microarray-based datasets, including GSE3167^16^ (14 cases of noncancerous bladder samples and 41 cases of bladder cancer samples), GSE13507^17^ (68 cases of noncancerous bladder samples and 165 cases of bladder cancer samples) and GSE19915^18^ (12 cases of noncancerous bladder samples and 143 cases of bladder cancer samples), were obtained from Gene Expression Omnibus (GEO, http://www.ncbi.nlm.nih.gov/geo/).

### Immunohistochemistry (IHC) analysis

All the specimens obtained from patients were fixed in 10% neutral formalin and then embedded by using paraffin. The embedded specimens were cut in 5-µm serial sections just before the staining. Immunohistochemistry was performed by using the SPlink Detection Kits SP-9000 (ZSGB-BIO, China) according to the manufacturer’s instructions. The sections processed by deparaffinization, rehydration and blocking endogenous peroxidase activity were incubated with primary antibody overnight at 4 °C. The primary antibodies used in the study include UBC9 (1:300) (sc-10759, Santa Cruz, CA) and IL6 (1:750) (ab9324 Abcam). After incubated with second antibody, a biotinylated donkey anti-goat IgG and horseradish peroxidase-labeled streptomycin working solution, the sections were treated with diaminobenzidine hydrochloride (DAB) to visualize the immunoreactivity. The immunohistochemical scoring was performed by two authors blind to the clinicopathological parameters independently. The score was composed of two parts. One was the extent of staining (percentage of positive tumor cells on a scale: 0% = 0; 1–25% = 1; 26–50% = 2; 51–75% = 3; 75–100% = 4), the other was the intensity of staining (no staining = 0; weak staining = 1; moderate staining = 2; strong staining = 3). The total scores ranged from 0 to 4 were considered as low expression, while ranged from 5 to 7 were considered as high expression.

### Cell culture and drug treatment

Human bladder cancer T24 and 5637 cells were obtained from the cell bank Shanghai Institute of Biochemistry and Cell biology, Shanghai, People’s Republic of China at November, 2013, which was less than 6 months before the start of the experiments. The cells were authenticated by using the short tandem repeat (STR) profiling method (Generi Biotech Ltd, Czech Republic). Last STR analysis was carried out 6 months before the start of the experiments. The cells were routinely grown in Dulbecco’s modified Eagle medium (DMEM), supplemented with 10% fetal bovine serum (FBS) at 37 °C in a humidified CO_2_ incubator (5% CO_2_). The SUMO inhibitor 2-D08 was purchased from Merck Millipore (Burlington, MA, USA)^14^. T24 and 5637 cells were treated with 50–100 μM concentrations of 2-D08 and control groups were treated with DMSO.

### Lentiviral vectors mediated UBC9-specific shRNA stable transfection

The shRNA targeting scrambled control and UBC9 were purchased from Transheep (Shanghai, China). The shRNA sequences were as the following: 5′-ccgg GAACTTCTAAATGAACCAAATCTCGAGATTTGGTTCATTTAGAAGTTCTTTTT g-3′. Target shUBC9 was first cloned into the pLKO.1 vector. And transfection was performed with Lipofectamine LTX reagent (Invitrogen) and Opti-MEM media. Lentivirus-transfected 293 T cells were incubated at 37 °C for 48 h and lentiviral particles were harvested by centrifugation and concentrated using the Lenti-Pac™ Kit (GeneCopoeia Inc., Guangzhou, China). Then T24 and 5637 cells were infected by concentrated virus and incubated for 48 h. Cells were selected with puromycin (2 μg/mL) 24 h later and cultivated 2 weeks to produce stable UBC9 shRNA-transfected vector.

Real-time quantitative polymerase chain reaction (RT-qPCR).

Total RNA was extracted using Trizol reagent (Invitrogen) according to the manufacturer’s instructions. cDNA was reverse-transcribed from 2 to 6 μg of the total RNA using M-MLV Reverse Transcriptase (Promega, Madison, WI). RT-qPCR were performed using StepOnePlus™ Real-Time PCR System (ABI, USA) in a volume of 20 μL reaction mixture including 0.1 μM primers, 10 μL 2 × FastStart Universal SYBR Green Master (Rox, Switzerland), and 20–100 ng cDNA sample. The primers used in the current study were list in Table S1. All experiments were repeated at least three times. The relative mRNA level was normalized using β-actin mRNA level and calculated using 2^−ΔΔCT^ method^19^.

### Western blot analysis

Total proteins were isolated using cell lysis buffer (Thermo, Rockford, IL, USA), followed by quantifying using BCA protein assay kit (Pierce, Rockford, IL, USA). About 50 μg protein was subjected to electrophoresis using SDS-PAGE, and then was transferred to the NC membrane. Membrane was incubated with antibody UBC9 (ab199073, Abcam) with a dilution of 1:1000 at 4 °C for 12 h. The antibody β-actin (ab8226, Abcam) was used as an internal control.

### Cell proliferation and migration assay

Cell proliferation was detected by methyl thiazolyl disphenyl tetrazolium bromide (MTT) assay and clone formation assay. For MTT assay, cells (0.5–1 × 103) were incubated for 24–120 h in 96-well tissue culture plates. After the incubation was over, we added 10 μL MTT stock solution (5 mg/mL; Sigma) into each well and incubation for 4 h at 37 °C. Then 100 μL dimethyl sulfoxide was added into each well. The absorbance was detected by using luminometer (FLUOstar Omega). For clone formation assay, 200, 400, and 800 cells were planted in 6-well plates and cultivated at 37 °C. The culturing was continued until the clones became visible by naked eyes. The clones were incubated with a staining solution containing 0.1% crystal violet and 20% methanol before being imaged and counted. Cell migration was estimated by scratch wound healing assay. Cells at a density of around 80% confluence were scratched by a sterile 200 µL plastic pipette tip. After 24 h, cells were fixed with methanol and photographed. ImageJ was used to quantitate the width of the wound.

### Apoptosis assay by annexin V-fluorescein isothiocyanate/propidium iodide (AnnexinV-FITC/PI) staining

Cells were harvested after 72 h transfection and washed using cold PBS. Then 100 µL of AnnexinV-FITC/PI was used to resuspend cells and incubate for 10–15 min. The FACSCalibur flow cytometry was used to examine stained cells.

### Cell cycle analysis (image-flow cytometry assay)

Transfected cells were harvested and washed with cold PBS twice follow by fixed in cold 70% ethanol. The Cell Cycle Analysis Kit (MultiSciences, China) was used to analyze the cell cycle distribution. The software ModFit 5.2 was used to sort and calculate the cells in different phases of cell cycle.

### Cytokine antibody arrays

The expression of cytokines in cells was detected by using Human Cytokine Antibody Array Membrane ab133998 (Abcam, Cambridge, UK) according to the manufacturer’s instructions. Briefly, cells were lysed and total protein was extracted. Then 200–250 μg of total protein was dissolved in blocking buffer and incubated with the array membrane at 4 °C overnight. The levels of cytokines were determined by chemiluminescence assay and quantified by densitometry, normalized to a positive control. This array membrane allows detecting 80 cytokines including: ENA-78, GCSF, GM-CSF, GRO, GRO-alpha, I-309, IL-1alpha, IL-1beta, IL-2, IL-3, IL-4, IL-5, IL-6, IL-7, IL-8, IL-10, IL-12 p40/p70, IL-13, IL-15, IFN-gamma, MCP-1, MCP-2, MCP-3, MCSF, MDC, MIG, MIP-1beta, MIP-1delta, RANTES, SCF, SDF-1, TARC, TGF-beta1, TNF-alpha, TNF-beta, EGF, IGF-I, Angiogenin, Oncostatin M, Thrombopoietin, VEGF-A, PDGF-BB, Leptin, BDNF, BLC, Ckß8-1, Eotaxin, Eotaxin-2, Eotaxin-3, FGF-4, FGF-6, FGF-7, FGF-9, Flt-3 Ligand, Fractalkine, GCP-2, GDNF, HGF, IGFBP-1, IGFBP-2, IGFBP-3, IGFBP-4, IL-16, IP-10, LIF, LIGHT, MCP-4, MIF, MIP-3 alpha, NAP-2, NT-3, NT-4, Osteopontin, Osteoprotegerin, PARC, PLGF, TGF-beta2, TGF-beta3, TIMP-1, TIMP-2^20^.

### RNA-seq analysis

Total RNA was extracted using Trizol reagent (Invitrogen). The construction of RNA-seq library was based on the protocol of the IlluminaTruSeq RNA Sample Preparation Kit (illumina). Finally, RNA-seq analysis was performed by WuXi NextCODE company (WuXi NextCODE, China) using Illumina HiSeqX Ten platforms. Each sample was repeated thrice. After quality control and trimming adaptor, reads were mapped onto human genome GRCh38 using the CLC Biomedical Genomics Workbench 4 (QIAGEN Bioinformatics), with the default mapping parameters. P < 0.05 and |fold change|> 2 were defined as the thresholds to identify differentially expressed genes (DEGs). The hierarchical clustering analysis was conducted by using pheatmap. Gene ontology (GO) enrichment analysis was performed by clusterProfiler^21^ and depicted using GOplot^22^. Gene Set Enrichment Analysis (GSEA) was conducted by GSEA software and visualized by using EnrichmentMap (Version 2.2.0) an app of Cytoscape^23^. The false discovery rate (FDR) less than 25% was considered as significance^24^. Protein–protein interaction networks (PPI) were constructed by STRING^25^ (https://string-db.org/) and visualized by using Cytoscape (Version 3.6.0)^26^. RNA-seq data have been deposited in the GEO database (GSE117143).

### Mass cytometry TOF (CyTOF)

Tissue samples were obtained from patients at First Affiliated Hospital of Guangxi Medical University. All solid tumor tissues were collected in DMEM media including 2% FBS (BI Biological), 1% penicillin–streptomycin (Life Technologies) and 0.2% fluconazole on ice. Complete media was prepared with 90% FBS (BI Biological) and 10% DMSO. The tumor tissues were each minced with scissors in 6 cm plates and digested in 2.5 mg/mL collagenase type II (Life Technologies) for 40 min at 37 °C. The cells were filtered through a 70 μm cell strainer, lysed in RBC buffer (Solarbio) , washed, resuspended in complete media and stored at − 80 °C. Resuspend cells to 3 × 10^6^/mL in Maxpar PBS (Fluidigm). Mix well and incubated in 1 mL of 5 μM cisplatin (Fluidigm) at room temperature for 5 min. Quench cisplatin staining by washing with Maxpar Cell Staining Buffer using 5 × the volume of the stained cells. Cells were fixed and permeabilized with 1 mL 1 × Fix I Buffer (Fluidigm) according to the manufacturer’s recommendations. Resuspend sample to be barcoded completely in 1 mL 1 × Barcode Perm Buffer (Fluidigm) and incubate samples for 30 min at room temperature. Following washed twice with Maxpar Cell Staining Buffer (Fluidigm). Cells were stained with an antibody cocktail for 30 min at room temperature. Cells were washed and fixed in 1.6% paraformaldehyde. Adding 1 mL of 125 nM Cell-ID Intercalator-Ir (191/193Ir, Fluidigm) to each sample and gently vortex. Incubate overnight at 4 °C. Wash cells with Maxpar Cell Staining Buffer and Milli-Q Water.Prepare 0.1 × EQ beads to re-suspend all samples, then filter through a 35-um nylon mesh and then analyzed on CyTOF 2 Mass Cytometer within the Fluidigm CyTOF acquisition software. CyTOF data were uploaded into Cytobank^27^, gating the populations of interest manually. To visualize the data in low dimension, the t-SNE algorithm was used. viSNE analysis was performed within the Cytobank web application, and viSNE plots were generated from each patient’ tumor samples.

### Statistical analysis

The statistical analysis was performed by using SPSS 20.0. Quantitative variable was summarized as means ± standard deviation (SD). The student’s t test was used to analyze the difference between two independent groups. Receiver operating characteristic (ROC) was constructed to evaluate the effectiveness of UBC9 to distinguish bladder cancer samples from noncancerous bladder cancer samples. Youden’s Index was used to identify the cut-off values. A value of P < 0.05 calculated by two-tailed test was considered significant.

## Results

### UBC9 up-regulated in bladder cancer

**Figure 1:**
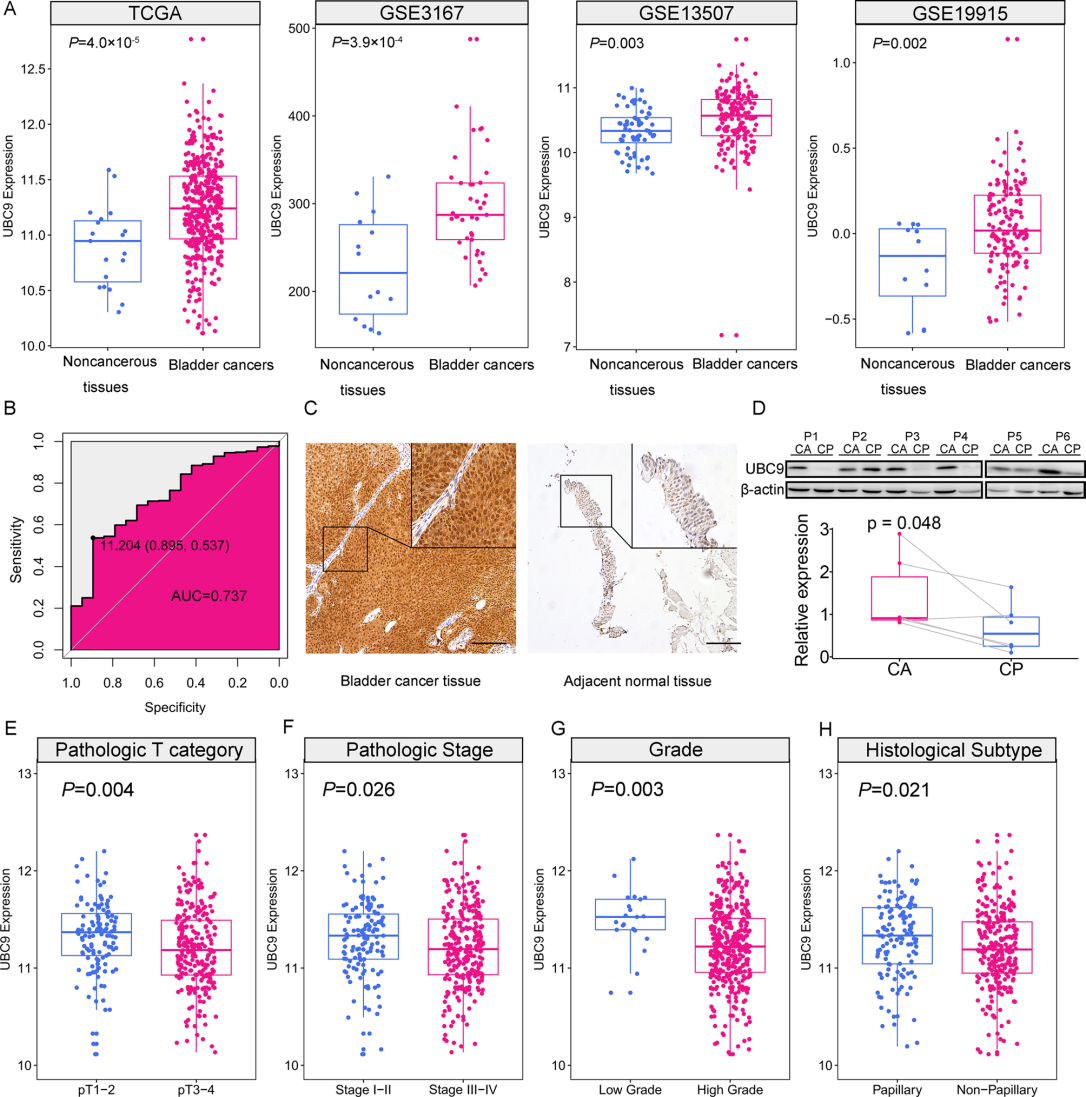
The expression and clinical significance of UBC9 in bladder cancer. (**A**) The expression of UBC9 in noncancerous bladder tissues and bladder cancer samples based on data from public datasets. (**B**) The ROC curve and AUC of UBC9. (**C**) Representative images of IHC detecting the expression of UBC9 in bladder cancer tissues and adjacent normal tissues. Scale bars = 20 μm. (**D**) Western blot detect UBC9 in 6 pairs of bladder cancer tissues and cancer-adjacent normal tissues. *CA* cancer tissues, *CP* cancer-adjacent normal tissues. (**E**) The expression of UBC9 in pathologic T1–T2 category vs. pathologic T3-T4 category. (**F**) The expression of UBC9 in pathologic stage i–ii vs. iii–iv. (**G**) The expression of UBC9 in low grade vs. high grade. (**H**) The expression of UBC9 in papillary vs. non- papillary. Means ± SD are shown. *IHC* immunohistochemistry, *ROC* receiver operator characteristic, *AU*C area under the curve.

To evaluate the expression of UBC9 in bladder cancer samples, we downloaded the bladder urothelial carcinoma TCGA data generated from 408 bladder cancer samples and 19 adjacent normal tissues. The TCGA data showed that UBC9 was remarkably upregulated in bladder cancer samples (P = 4.0 × 10^–5^). Datasets from GEO also demonstrated that UBC9 was significantly increased in bladder cancer samples (P = 3.92 × 10^–4^ for GSE3167, P = 0.003 for GSE13507, P = 0.002 for GSE19915) (Fig. 1A). To assess the diagnostic value of UBC9 in bladder cancer, the receiver operator characteristic (ROC) curve was employed to analyze the sensitivity and specificity based on TCGA data. The area under the curve (AUC) of UBC9 was 0.737. And the sensitivity and specificity at a cut-off value of 11.20 were 0.54 and 0.90, respectively (Fig. 1B).

To further validate the results from public datasets, we performed IHC staining in 106 bladder cancer samples and 14 adjacent normal tissues. UBC9 was detected in both nucleus and cytoplasm and a high level of UBC9 in nucleus was, in most cases, associated with a high level of UBC9 in cytoplasm. So, the UBC9 score in the present study was a combination of both nuclear and cytoplasmic staining signal. The expression of UBC9 was significantly higher in bladder cancer samples (84.9%, 90/106) compared with adjacent normal tissues (42.9%, 6/14) (P = 0.001) (Fig. 1C, Table S2). To further analyze the expression of UBC9 protein, we used western blot to detect UBC9 in another 6 pairs of bladder cancer tissues and adjacent normal tissues. The results showed that UBC9 protein was up-regulated in cancer tissues (Figs. 1D and S1, P = 0.048).

To explore the relationship between UBC9 expression and clinicopathological features, we stratified patients according to different clinicopathological parameters and compared the expression of UBC9. The expression of UBC9 was notably higher in pT1-2 than that of pT3-4 (Fig. 1E, P = 0.004). With regard to TNM stage, the expression of UBC9 in advanced stage patients (III and IV) was lower compared with early stage (I and II) (Fig. 1F, P = 0.026). Compared with those in high grade, the level of UBC9 in patients with low grade was significantly higher (Fig. 1G, P = 0.003). As for the histological subtype of the bladder cancer samples, we found that the expression of UBC9 was significantly higher in papillary than in non-papillary subtype (Fig. 1H, P = 0.021).

### Knocking-down of UBC9 inhibits cell proliferation and arrests cell cycle progression

**Figure 2:**
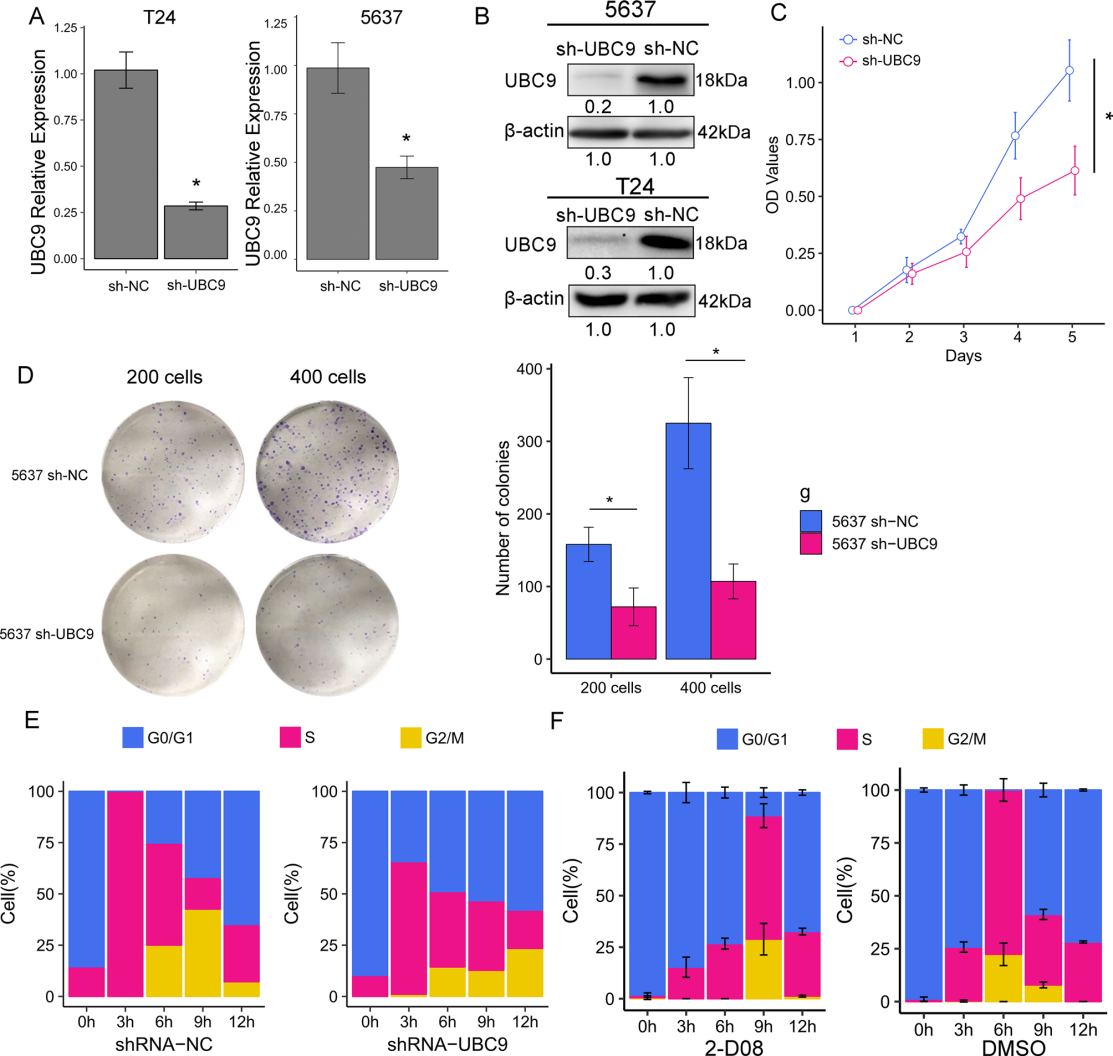
Knockdown of UBC9 inhibits proliferation andarrests cell cycle progression in bladder cancer cell. (**A**) RT-qPCR detected the expression of UBC9 in cells transfected with shRNA-NC and shRNA-UBC9. (n = 3 independent preparations) (**B**) Western blot detected the expression of UBC9 in cells transfected with shRNA-NC and shRNA-UBC9. (**C**) Effect of silencing UBC9 on cell proliferation evaluated by MTT assay. (n = 3 independent preparations) *P < 0.05. (**D**) Clones were stained with Giemsa and photographed with a digital camera. The number of clones was accurately calculated and statistically analyzed. (**E**) The cell cycle was synchronized using a double thymidine block. The stacked plots showed the percentage of the cells in each cycle phase at each time point. Left: cells transfected with shRNA-NC. Right: cells transfected with shRNA-UBC9. Blue: G0 phase. Red: S phase. Gold: G2 and M phase. (**F**) The cell cycle was synchronized using a double thymidine block. The stacked plots showed the percentage of the cells in each cycle phase at each time point. Left: cells transfected with 2-D08. Right: cells transfected with DMSO.

To explore the biological function of UBC9 in bladder cancer, we established two bladder cancer cell lines, T24 and 5637, with stable expression of shRNA targeting UBC9 (shRNA-UBC9) and negative control shRNA (shRNA-NC). The effect of knockdown was confirmed by using RT-qPCR and western blot. As shown in Fig. 2A, the UBC9 mRNA expression was significantly decreased after shRNA-UBC9 transfection (T24: P = 0.009; 5637: P = 0.003). The relative mRNA expression was reduced by 72.2% and 50.3% in T24 and 5637 cells, respectively, compared to the control group. Furthermore, the UBC9 protein levels were also downregulated after knockdown of UBC9 (Figs. 2B, S2). These results indicated the efficient knockdown of UBC9.

To address the function of UBC9 in bladder cancer cells, we first compared the cell proliferation between shRNA-UBC9 and shRNA-NC group by using MTT assay in T24 cells. We found that knockdown of UBC9 significantly decreased cell proliferation (P = 0.030) (Fig. 2C). In clone formation assay, we also found that the colony forming abilities of 5637 cells was significantly inhibited compared to negative control cells (Fig. 2D). To determine the biological role of UBC9 in cell cycle, we compared cell cycle progression between shRNA-UBC9 group and shRNA-NC group in T24 cells dynamically. The cell cycle was firstly synchronized at the G1/S boundary by a double thymidine block and then released in the same time. With the cell cycle progression, we found that, in the shRNA-NC group, most of the cells in G0/G1 phase passed S phase and then entered G2/M phase in 6–12 h. However, in the shRNA-UBC9 group, cells in the G0/G1 phase entered S phase slowly in 0–12 h, suggesting that knockdown of UBC9 caused a cell cycle arrest in G0/S phase (Fig. 2E). To further validate the effect of UBC9 in cell cycle, we treated T24 cells with 50 μM 2-D08, which inhibits sumoylation by blocking transfer of SUMO from the UBC9-SUMO thioester to the substrate^14^. As shown in Fig. 2F, T24 cells treated with 2-D08 remained in G0/G1 phase in 0–6 h, indicating 2-D08 arrested cell cycle progression in G1/S phase. The representative flow charts were shown in Fig. S3.

### Knocking-down of UBC9 inhibits cell migration and promotes cell apoptosis

**Figure 3:**
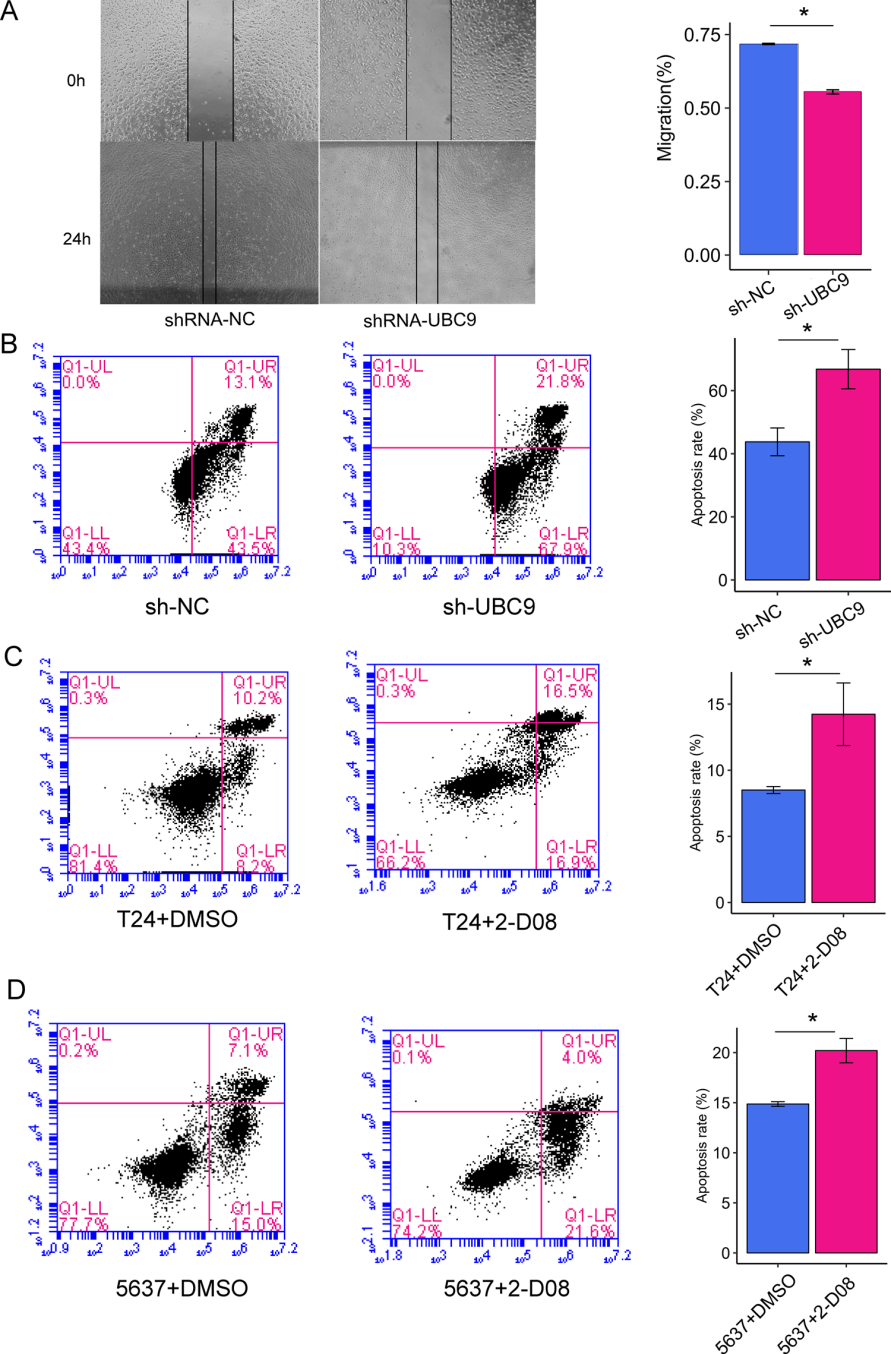
Silencing of UBC9 inhibits migration and promotes apoptosis. (**A**) Representative images of wound healing assay at 0 h and 24 h and quantization of cell migration in the wound healing assay during 24 h. (n = 3 independent preparations). (**B**–**D**) Effect of silencing UBC9 on cell apoptosis evaluated by AnnexinV-FITC/PI staining. The right upper quadrant showed the percentage of later apoptotic cells and the right lower quadrant showed the percentage of early apoptotic cells. Means ± SD are shown.

Given that cell migration is a vital step in cancer progression and metastasis, we performed a scratch wound healing assay to evaluate the role of UBC9 in bladder cancer cell migration. As shown in Fig. 3A, the wound closure of shRNA-UBC9 group was significantly delayed after 24 h (Fig. 3A, P = 0.001). To evaluate the function of UBC9 in cell apoptosis, we used a flow cytometer to compare the apoptosis rate between the two groups. The proportion of early apoptotic cells was significantly increased in the shRNA-UBC9 compared with the shRNA-NC group (66.8% vs. 43.8%, P = 0.006) (Fig. 3B). In addition, we observed similar results by using SUMOylation inhibitor 2-D08. T24 cells and 5637 cells were treated with 100 μM 2-D08 respectively. As shown in Fig. 3C,D, the early apoptotic cells were significantly increased in T24 cells and 5637 cells treated with 2-D08 (T24: P = 0.014; 5637: P = 0.014). These results indicated that silencing of UBC9 inhibited migration and promoted apoptosis in bladder cancer cells.

### UBC9 regulates inflammation-related pathways in bladder cancer

**Figure 4:**
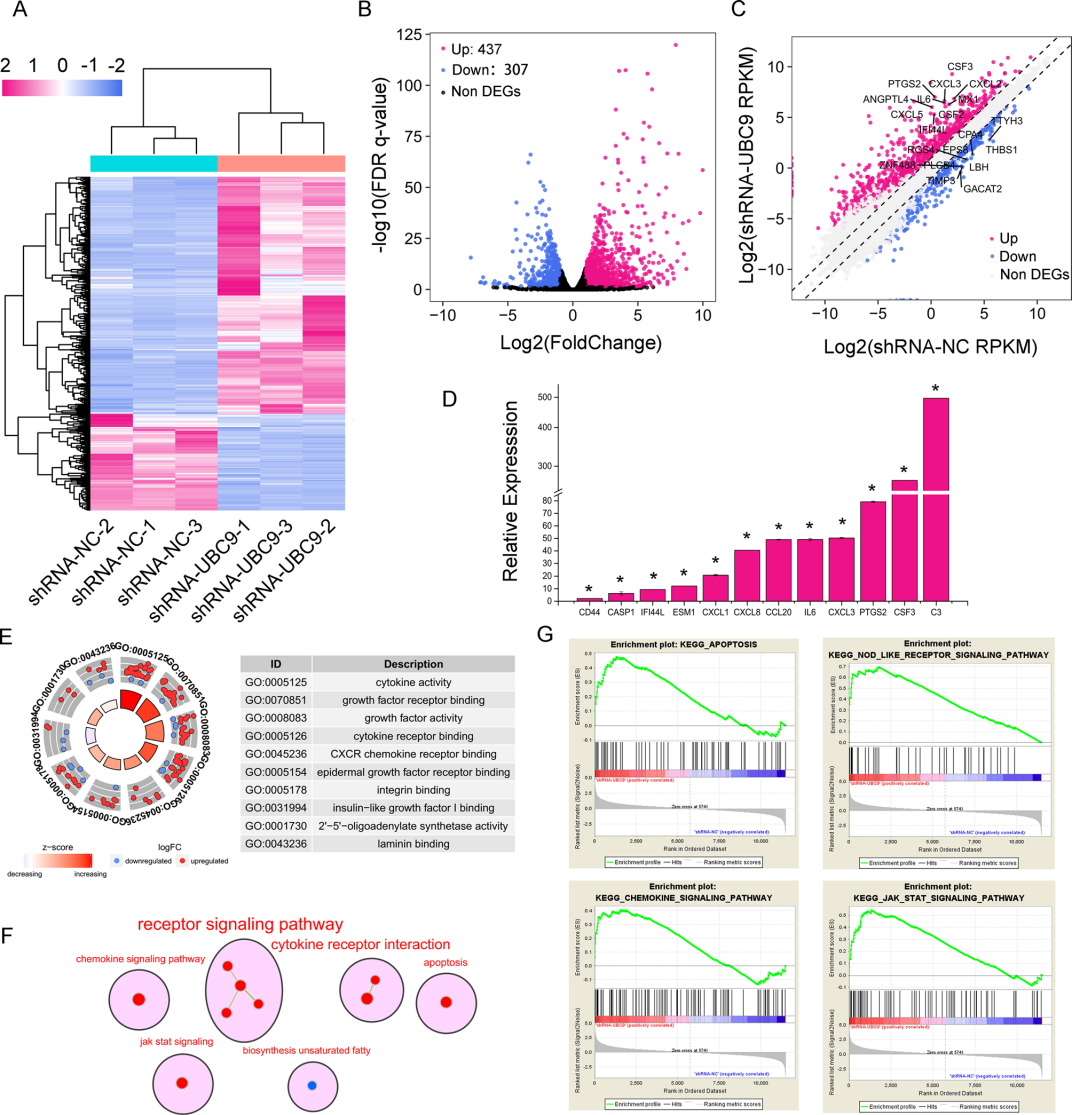
Differentially expressed genes and enrichment analysis after knockdown of UBC9. (**A**) Heat map showed the expression of DEGs in each replicate. Samples (column) and genes (row) were clustered by unsupervised hierarchical cluster analysis. (**B**) Volcano plots showed the differential expression of genes between shRNA-UBC9 and shRNA-NC. Red dots represented the significantly upregulated genes in shRNA-UBC9 group compared with shRNA-NC group (Log2(foldchange) > 1 & FDR P < 0.05). Blue dots represented the significantly downregulated genes in shRNA-UBC9 group compared with shRNA-NC group (Log2(foldchange) < − 1 & FDR P < 0.05). Black dots represented non DEGs. (**C**) Scatterplot illustrated the expression of genes in the shRNA-UBC9 group and shRNA-NC group. The top 10 upregulated and top 10 downregulated genes with RPKM over 0.5 in shRNA-UBC9 group were marked. Reference line represented twofold change. (**D**) The expression of selected DEGs was validated by RT-qPCR. Data was generated from three independent studies. Means ± SD are shown. *P < 0.001. (**E**) The GOCircle plot showed the top 10 significant GO molecular function terms based on adjust P-value. The height of the bar in the inner ring suggested the significance of the corresponding term (− log10 adjusted P-value), and color corresponded to the z-score. The scatterplots plot in the out ring showed the expression level of genes (logFoldChange) in the corresponding term. The term IDs in the out ring corresponded to the description in the right table. (**F**) The map illustrated the results of GSEA. Red dots represented the pathways significantly upregulated in shRNA-UBC9 group. Blue dots represented the pathways significantly downregulated in shRNA-UBC9 group. Clusters of functionally related gene-sets were circled and assigned a representative label. (**G**) The top 4 most significant pathways identified by GSEA.

To identify genes potentially regulated by UBC9 in bladder cancer, RNA-seq was performed on shRNA-UBC9 and shRNA-NC T24 cells, with each sample repeated three times. DEGs were selected based on the cutoff value of |log (fold change)|> 1 and FDR < 0.05. Unsupervised hierarchical cluster analysis indicated that the three replicates of each group were highly concordant (Fig. 4A). Totally, 744 DEGs including 437 up-regulated genes and 307 down-regulated genes were identified (Fig. 4B). The top 10 upregulated and top 10 down-regulated genes with RPKM over 0.5 in shRNA-UBC9 group were marked in Fig. 4C. Unexpectedly, we observed that many of the significant up-regulated genes were inflammatory factors. The CSF3, PTGS2, CXCL3, IL6 and CCL20 were the top five most increased genes. To validate the results of RNA-seq, we selected 11 DEGs to detect their expression using RT-qPCR. As shown in Fig. 4D, the results generated by RT-qPCR were similar to those detected by RNA-seq, suggesting the credibility of RNA-seq. To further validate these results, we used RT-qPCR to detect the expression of these genes in another bladder cancer cell line, 5637 cells, after knockdown of UBC9. As shown in Fig. S4A, the expression levels of IF44IL, ESM1, CXCL1, CXCL8, CCL20, PTGS2, CSCF3 and IL6 were significantly increased after knockdown of UBC9. Besides, we observed similar results in 5637 cells that the expression levels of CD44, CXCL1, PTGS2, CXCL8, CSF3, ESM1, CXCL3 and IL6 were significantly increased after being treated with SUMOylation inhibitor 2-D08. (Fig. S4B). These results indicated that knockdown of UBC9 triggered dramatic inflammation in bladder cancer.

**Figure 5:**
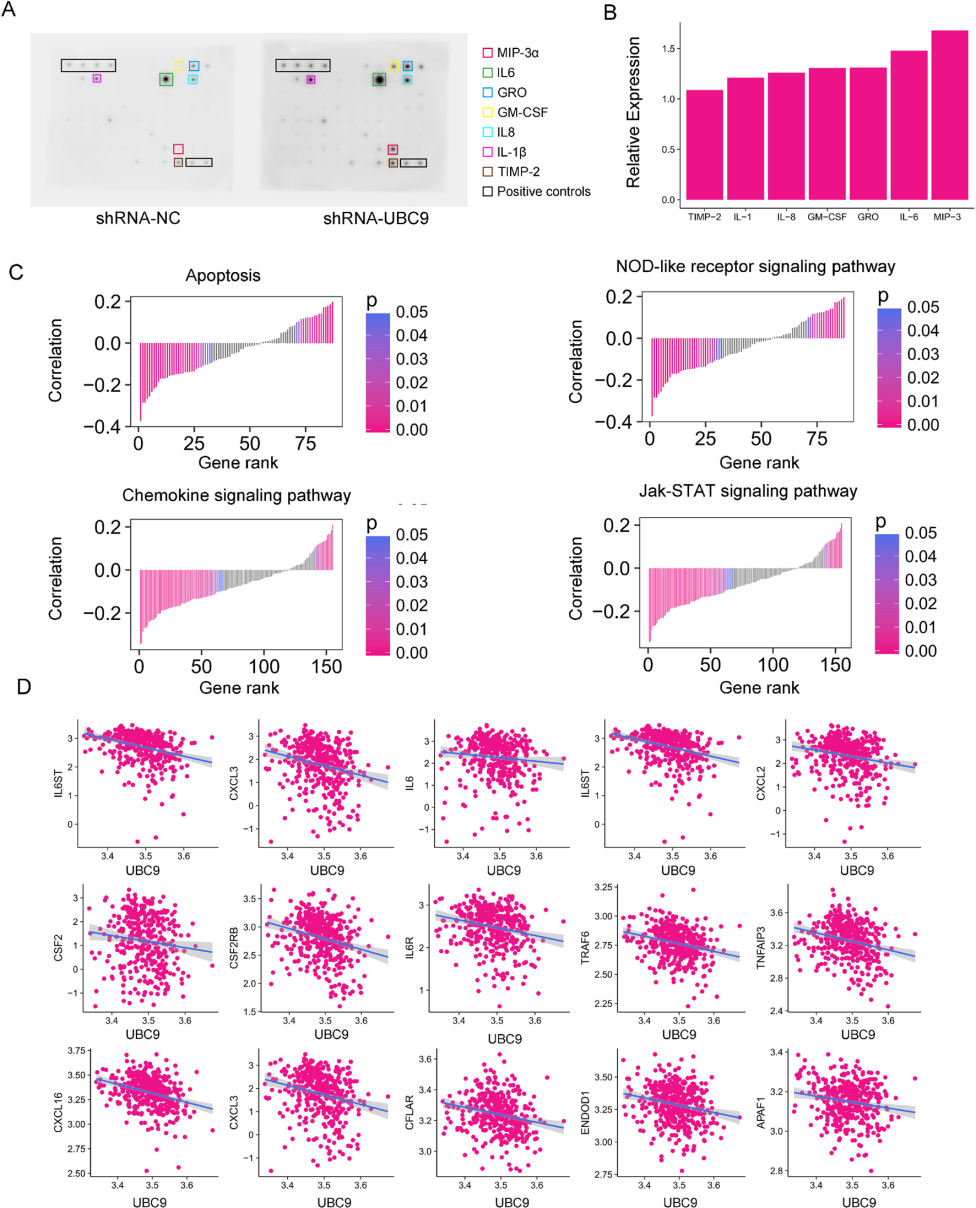
UBC9 regulates inflammatory and apoptotic pathways in bladder cancer. (**A**) The images of antibody arrays of shRNA-NC (left) and shRNA-UBC9 (right). The significantly differential expression cytokines were marked by different colored squares. (**B**) Normalized expression of upregulated cytokines in shRNA-UBC9 detected by antibody arrays. (**C**) The correlation between UBC9 level and the expression of genes in apoptosis, NOD-like receptor signaling pathway, chemokine signaling pathway and Jak-STAT signaling pathway (TCGA data). (**D**) The correlation between UBC9 levels and key genes in the above-mentioned pathways.

To gain insights into the regulatory mechanisms of UBC9 in bladder cancer, we performed GO molecular function enrichment analysis by using clusterProfiler. The DEGs served as input data and the top ten most significant GO terms were shown in Fig. 4E. The cytokine activity (GO:0005125) was the most significant term with an adjust P-value of 5.46 × 10^–5^. We observed that most of the genes enriched in cytokine activity were upregulated after UBC9 silencing. Furthermore, cytokine activity, cytokine receptor binding and CXCR chemokine receptor binding were closely related with inflammation, thus, knockdown of UBC9 triggered dramatic inflammation in bladder cancer cell. To examine the possible pathways regulated by UBC9 in bladder cancer, we conducted GSEA using the KEGG dataset from the Molecular Signatures Database. Most of the significantly upregulated pathways in shRNA-UBC9 group were related with inflammation. The biosynthesis unsaturated fatty pathway was the only significantly enriched pathway that downregulated in shRNA-UBC9 group (Fig. 4F). In the upregulated pathways, the apoptosis, NOD-like receptor signaling pathway, chemokine signaling pathway and Jak-STAT signaling pathway were the top four significant pathways (Fig. 4G). These results suggested that knockdown of UBC9 caused significantly active inflammation and apoptosis. To further validate the results, we employed a human cytokine antibody arrays to detect the expression of cytokine in protein level. As shown in Fig. 5A and B, the expression of MIP-3α, IL-6, GRO, GM-CSF, IL-8, IL-1β and TIMP-2 were increased after UBC9 knockdown. Interestingly, in bladder cancer simples, most of the genes in the apoptosis, NOD-like receptor signaling pathway, chemokine signaling pathway and Jak-STAT signaling pathway were up-regulated when UBC9 was down-regulated (Fig. 5C). We found that many key molecules in the above-mentioned pathways were significantly negatively relative with UBC9, such as IL6ST (r = − 0.34, P = 2.68 × 10^–12^), CXCL16 (r = − 0.33, P = 6.68 × 10^–11^), IL6 (r = − 0.17, P = 6.97 × 10^–4^) and JAK1(r = − 0.20, P = 4.82 × 10^–5^) (Fig. 5D). These results indicated UBC9 was critical to homeostasis and modulation of cellular response under stress, without the protection of UBC9, bladder cancer cells suffered from intense inflammation.

### Inflammation and cancer stem cell-like population increased in UBC9-low bladder cancer samples

**Figure 6:**
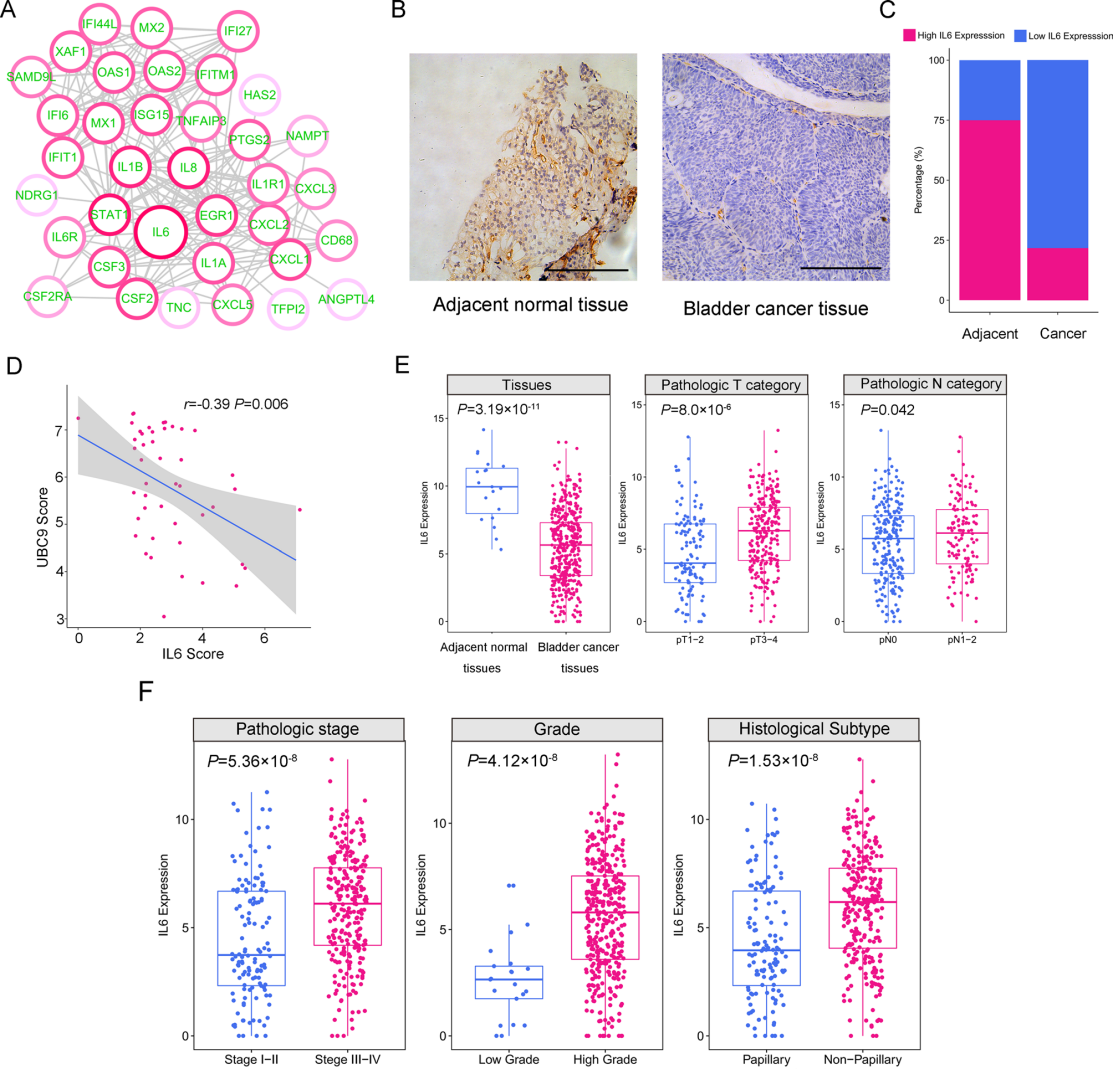
IL6 is located in the hub of the regulatory network of UBC9. (**A**) The protein‑protein interaction (PPI) network of DEGs. The red circles represent the up-regulated genes in the shRNA-UBC9. A darker color indicated a greater difference in gene expression. The IL6 was the hub in the network. (**B**) Representative images of IHC detecting the expression of IL6 in bladder cancer samples and adjacent normal tissues. Scale bars = 20 μm. (**C**) The expression of IL6 in bladder cancer samples and adjacent normal tissues detected by IHC (n of bladder cancer samples = 46, n of adjacent normal tissues = 8). (**D**) The correlation between UBC9 levels and IL6 levels based on immunohistochemical scores. (**E**–**F**) The correlation between IL6 levels and clinical parameters (TCGA).

**Figure 7:**
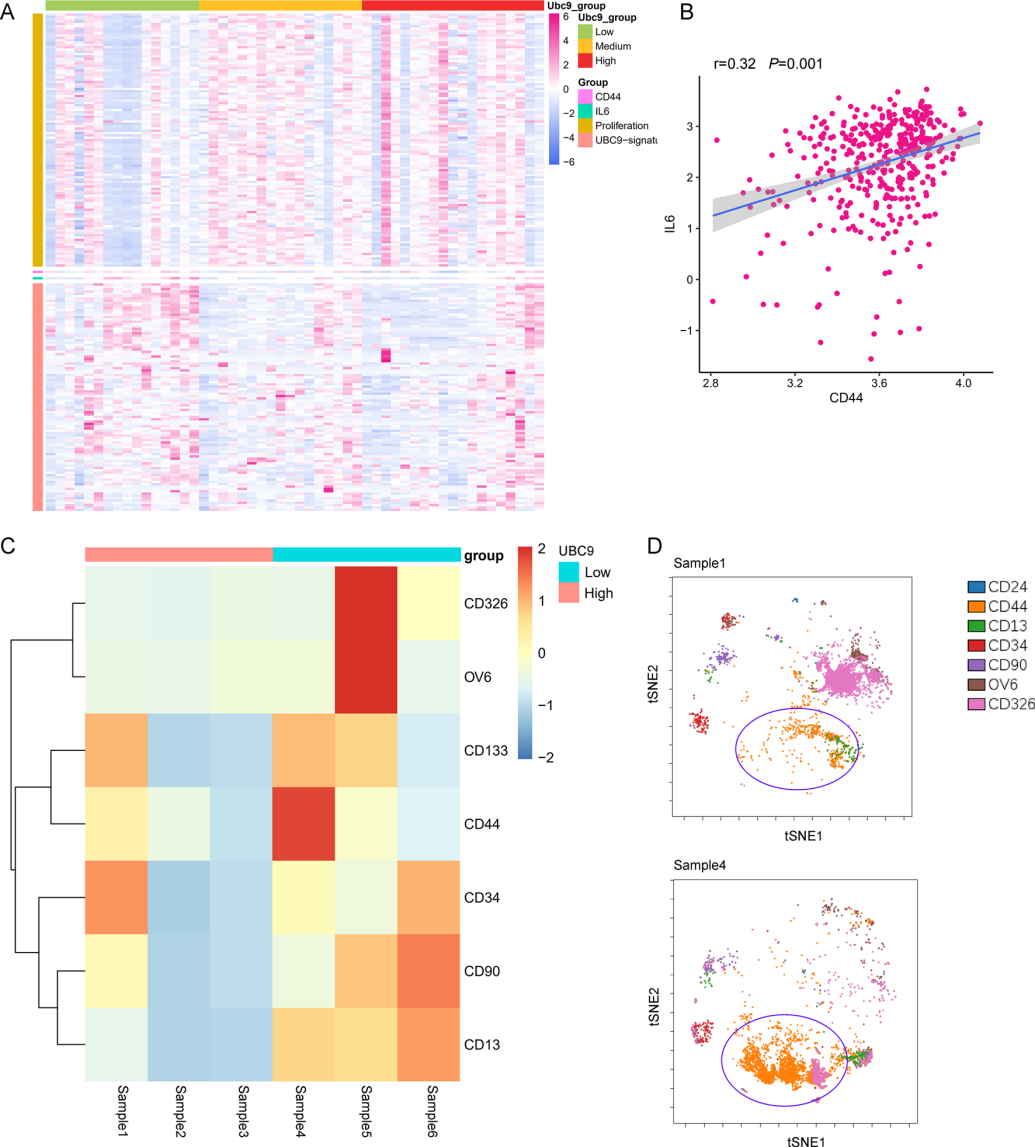
Inflammation and cancer stem cell-like population increased in UBC9-low bladder cancer samples. (**A**) Heatmaps of UBC9-signature and proliferation-signature^28^. 52 case of bladder cancer samples were divided into high, medium and low groups according to the mRNA expression of UBC9. proliferation-signature, UBC9-signature CD44 and IL6 were shown. (**B**) The correlation between IL6 levels and CD44 levels. (**C**) Heatmap of mean level cancer stem cells markers. Samples were divided into two groups according to the expression of UBC9 and the expression of cancer stem cells markers were detected by CyTOF. (**D**) Representative tSNE plot from sample1 (UBC9-up) and sample4 (UBC9-down). The blue circle indicated the CD44-positive cell population.

Since proteins seldom take effect in isolation, it is important to know the interactions between the DEGs and identify the hub gene in the UBC9-regulated network. We constructed the PPI network by using STRING database. The PPI network contained 37 nodes and 211 edges and the average node degree was 11. The IL6 with the highest degree of 23 was considered as a hub gene in the UBC9-regulated network (Fig. 6A). To determine the expression of IL6 in bladder cancer samples, we performed IHC in 46 bladder cancer samples and 8 adjacent normal samples. The expression of IL6 was significantly lower in bladder cancer samples (21.7%, 10/46) compared with adjacent normal tissues (75.0%, 6/8) (P = 0.009) (Fig. 6B,C). The negative correlation between UBC9 levels and IL6 levels was also validated the based on IHC (P = 0.006, Fig. 6D). To evaluate the clinical significance of IL6 in bladder cancer, we analyzed the relationship between IL6 and clinical parameters based on TCGA dataset. We also found that IL6 was significantly downregulated in cancer tissues (P = 3.19 × 10^–11^, Fig. 6E). But, the levels of IL6 was significantly increased in patients with pT3-4, lymphatic metastasis, advanced stage, high-grade, non-papillary of gross appearance (all P < 0.05, Fig. 6E,F), which were exactly opposite to UBC9. We further investigated the relationship between IL6 and UBC9 in bladder cancer samples. 52 case of bladder cancer samples were divided into high, medium and low groups according to the mRNA expression of UBC9. A UBC9-signature was generated, which consist of 98 increased inflammation-related genes after knockdown of UBC9 in T24 cells. In addition, a proliferation-signature was collected from literature^28^. As shown in Fig. 7A, the expression of UBC9-signature was increased in the low group compared with that in the medium and high groups. Inversely, the proliferation-signature was more intense in the high group compared with low group. We also observed that IL6 was increased in the low group. These results indicated that low expression of UBC9 in bladder cancer was associated with activated inflammation and IL6, while high expression of UBC9 was associated with proliferation. IL6-Jak/STAT signaling pathway is implicated in the stemness maintenance of bladder cancer^29^. Interestingly, the expression of CD44, a recognized marker of bladder cancer stem cells^30^, increased two times after UBC9 knockdown (Fig. 4D). We analyzed the correlation between IL6 and CD44. CD44 was positively related with IL6 (Fig. 7B, P = 0.001). CD44 is a widely accepted marker of bladder cancer stem cells, we employed an emerging technology, mass cytometry TOF (CyTOF) which is novel single-cell analysis technology with the ability to analyze more than 35 unique parameters on a million cells, to analyze the cancer stem cell-like population between UBC9-high and UBC9-low bladder cancers samples. We collected 6 bladder cancer samples and divided them into 2 groups according to the expression of UBC9 detected by RT-qPCR. We used CyTOF with a panel of 7 CSCs markers was used for an analysis of how the cancer stem cell-like populations changed with UBC9 expression. As shown in Fig. 7C, most of the CSCs markers were up-regulated in the UBC9-low group. And the CD44-positve cell population was increased in the UBC9-low group (Fig. 7D). These results indicated that low expression of UBC9 might be associated with the stemness maintenance of bladder cancer.

### Epithelial–mesenchymal transition was activated in UBC9-low bladder cancer

**Figure 8:**
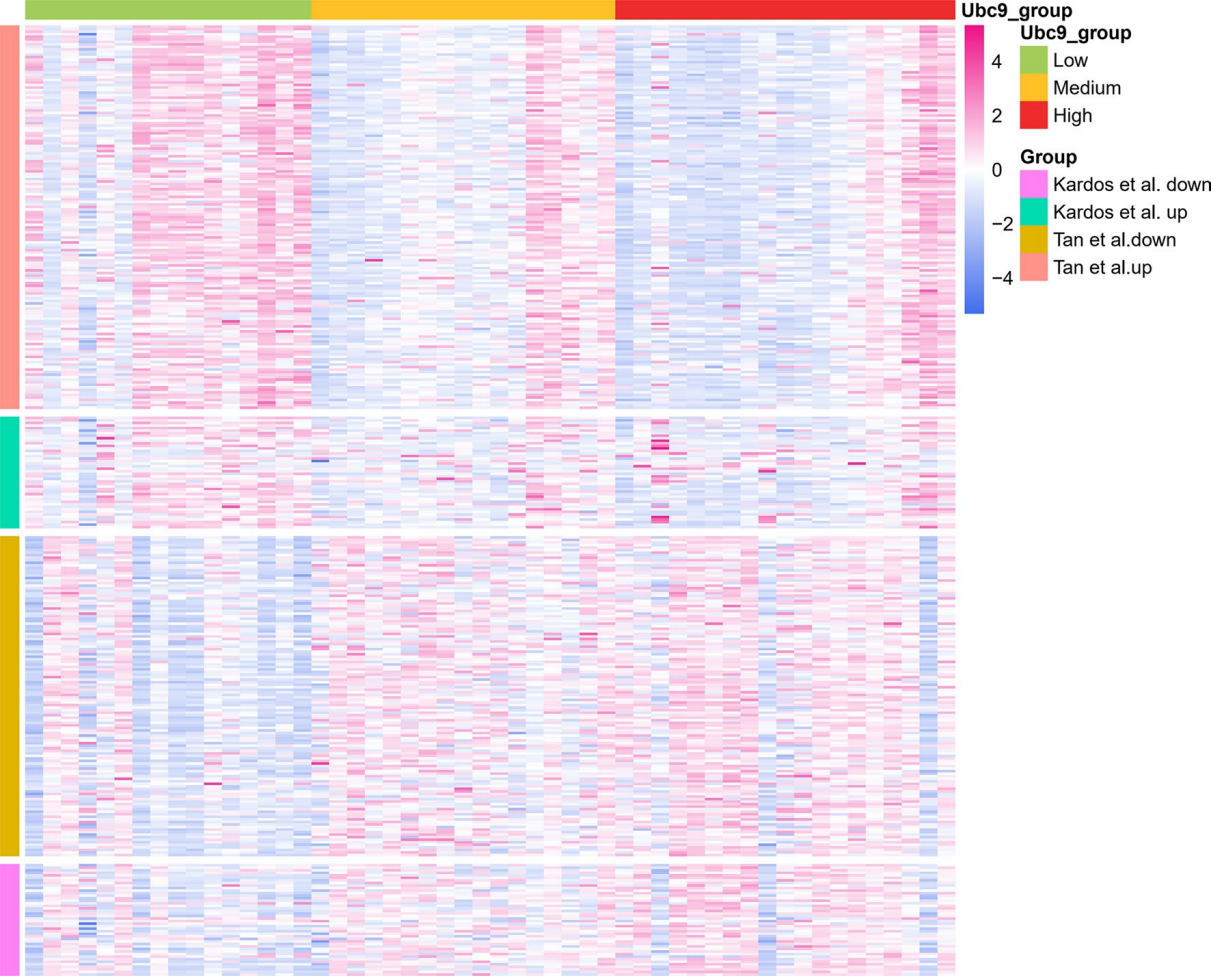
Heatmaps of two bidirectional EMT signatures. 52 case of bladder cancer samples were divided into high, medium and low groups according to the mRNA expression of UBC9. An EMT signatures was collected from literature^32^.

Epithelial–mesenchymal transition (EMT) promotes the gain of stem cell characteristics and sustains stem cell‐like populations^31^. During the process of EMT, cancer cells lose their epithelial morphology and adopt a spindle‐shaped and mesenchymal appearance progressively. Inflammation is an important trigger of EMT. Given that the key function of UBC9 in alleviating inflammation, we investigated whether UBC9 was associated with activated EMT in cancer samples. An EMT-signature in bladder cancer was collected from literature^32^. The Up-signature consist of up-regulated gene markers in the mesenchymal phenotype while the down-signature was decreased in mesenchymal phenotype. We observed that up-signature was increased but down-signature was decreased in UBC9-low group (Fig. 8). These results indicated that low expression of UBC9 in bladder cancer was associated with EMT.

## Discussion

It is widely accepted that UBC9, a hallmark of activated sumoylation, plays a vital role in maintaining the homeostasis and restraining stress responses. Inflammation is also a kind of stress responses. Hypoxia stress, oxidative stress, cytokines stimulation, chemotherapy drugs and activated oncogenes induce inflammatory reaction in cancer cells^8^. Intense inflammation induces the production of reactive nitrogen species (RONS), which causes cellular senescence, suppresses tumor proliferation and leads to apoptosis^33,34^. However, inflammation is the origin of cancer. Inflammation leads to increased DNA mutation rates and overall genetic instability; free radicals suppress the activity of DNA mismatch repair genes and increased chemokines contribute to tumor growth and angiogenesis^35,36^. In the present study, we reported a dual role of UBC9 in bladder cancer. UBC9 in bladder cancer cells is required to maintain high sumoylation levels and alleviate stress-related inflammation threats to cell survival. In contrast, low expressed UBC9 in bladder cancer might contribute to activation of inflammation, stem cell-like population formation and EMT.

Both public data and our in vitro experiments showed that UBC9 expression was elevated in bladder cancer samples compared to adjacent tissues. However, we observed that UBC9 expression was down-regulated with tumor progression. Knockdown of UBC9 inhibited cell proliferation, cell cycle progression, cell migration and promoting apoptosis. Inflammatory pathways were remarkably active after knockdown of UBC9. Our results are in line with previous research showing that Ubc9^−/−^ accompanied by impaired sumoylation exhibited globally heightened inflammation^37^. UBC9 knockdown clearly activates inflammation-related and apoptosis pathways. Our examination of bladder cancer samples revealed UBC9 expression was negatively correlated with most genes in pathways linked to chemokine signaling, apoptosis, NOD-like receptor signaling, and Jak-STAT signaling. Thus, these results suggested that bladder cancer cells required UBC9 to restrict inflammation. Without the protection of UBC9, cancer cells would suffer from a cytokine storm, resulting in compromised migration and increased apoptosis. Thus, UBC9 may not be a “driver” but a “protector” of bladder cancer tumorigenesis.

IL6 was a key component in the regulatory network of UBC9. The expression of IL-6 increased 17-fold after UBC9 knocking-down. IL-6, one of the major cytokines, is deregulated in cancer^38^. IL6 overexpression can reduce cell proliferation, migration, and invasion both in vitro and in vivo^39^. In the present study, we found that IL6 expression was downregulated in bladder cancer samples, but upregulated in advanced cancer stages and grades compared with adjacent non-malignant tissues, exactly opposite of UBC9. In recent years, IL6 has attracted widespread attention as a double-acting inflammatory factor with pro- and anti-inflammatory roles^38,40^. High IL6 expression in bladder cancer samples has also been associated with advanced stages, higher post-treatment recurrence rate, and decreased survival rate. Moreover, blocking IL6 expression attenuated invasive capability of bladder cancer cells^41^. The exact mechanism of IL6 in bladder cancer is still unclear^42^. IL6 might increase oncogene expression through activating the Janus kinase/signal transducer and activator of transcription (Jak-STAT) signaling pathway^40^. Previous research indicated that IL6-STAT3 signaling contributed to the induction of CD44^43,44^, a prominent marker of cancer stem cells in various malignancies, including bladder cancer. We found that CD44 was upregulated and positively correlated with IL6 after UBC9 knockdown. And CyTOF indicated that CD44-positive stem cell-like population was increased. These results led us to speculate that low expression of IL6 levels appear to benefit tumor growth, while high expression of IL6 activates inflammation and tumor stem cells, facilitating cancer progression.

UBC9 is emerging as a novel cancer therapeutic target for enhancing chemosensitivity and inducing synthetic lethality^45^. However, based on the findings of this study, we caution that pharmacological impairment of UBC9 may have unexpected risks. Instead of damaging tumor cells, attenuated UBC9 may activate inflammatory pathways and thus facilitate cancer progression. Context-specific studies with more refinement are needed before UBC9 can be safely used in cancer treatments.

## Conclusions

This study is the first to focus on UBC9 function in bladder cancer. We demonstrated that UBC9 was up-regulated in bladder cancer samples. Knockdown of UBC9 inhibited proliferation, arrested cell cycle progression and promoted apoptosis in bladder cancer cells. But attenuated UBC9 activated inflammatory pathways and increased cancer stem cell-like population. We therefore concluded that UBC9 might be required to maintain high sumoylation levels and alleviate stress-related inflammation threats to cell survival, providing a promising candidate for targeted therapy.

## Supplementary information

Supplementary Information.

## Data Availability

The datasets generated and/or analysed during the current study are available in the [Gene Expression Omnibus (GEO)] repository, [https://www.ncbi.nlm.nih.gov/geo (accession no. GSE117143)].

## Abbreviations

UBC9: Ubiquitin-conjugating enzyme 9
SUMO: Small ubiquitin-related modifier
