# Supplementary material for: UBC9 coordinates inflammation affecting development of bladder cancer
Source: Sci Rep. 2020 Nov 26;10:20670. doi: 10.1038/s41598-020-77623-9 (PMC7691338; doi:10.1038/s41598-020-77623-9)
Supplement: Supplementary file 1 — Supplementary Information. [file 41598_2020_77623_MOESM1_ESM.pdf]

UBC9 coordinates inflammation affecting development of bladder cancer

Xiaoliang Huang[2,3,4†], Yuting Tao[2,3,4†], Jiamin Gao[2,3,4], Xianguo Zhou[2,3,4], Shaomei Tang[2,3,4], Caiwang Deng[2,3,4], Zhiyong Lai[2,3,4], Xinggu Lin[2,3], Qiuyan Wang[2,3,4*], Tianyu Li[1,2,3*]

1 Department of Urology and Nephrology, the First Affiliated Hospital of Guangxi Medical University, Nanning, China

2 Center for Genomic and Personalized Medicine, Guangxi Medical University, Nanning, Guangxi Zhuang Autonomous Region, China

3 Guangxi key laboratory for genomic and personalized medicine, Guangxi, collaborative innovation center for genomic and personalized medicine, Nanning, Guangxi Zhuang Autonomous Region, China

4 Center for Translational Medicine, Guangxi Medical University, Nanning, Guangxi Zhuang Autonomous Region, China

* Correspondence: Tianyu Li, Tel: +86 7715353342; Fax: +86 7715353342; Email: 547370852@qq.com; & Qiuyan Wang, Tel: +86 7715353342; Fax: +86 7715353342; Email: qiuyanwang510@yahoo.com;

† These authors contributed equally to this work.

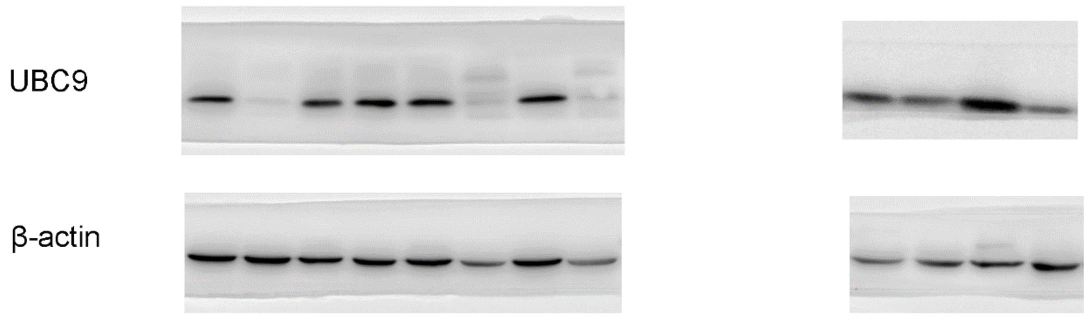

Figure S1: The original western blot membranes for membrane strips presented in Figure 1D.

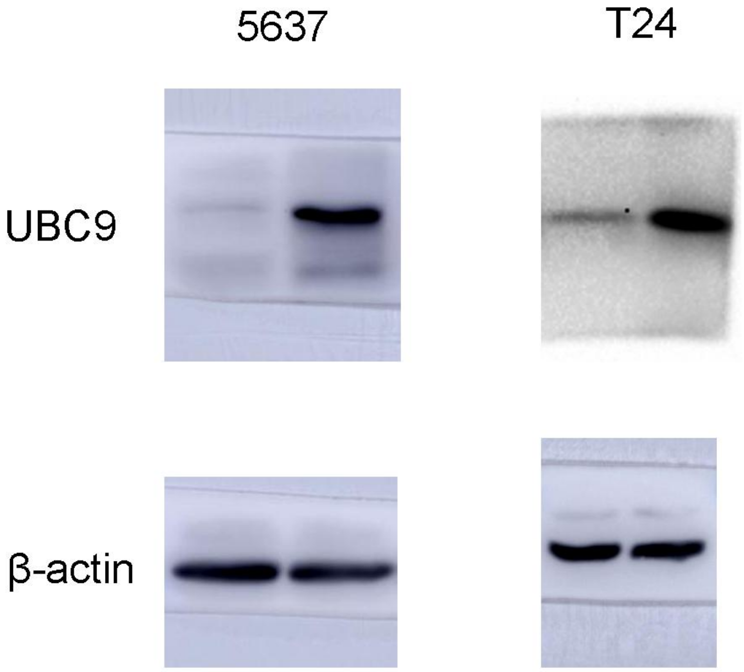

Figure S2: The original western blot membranes for membrane strips presented in Figure 2B.

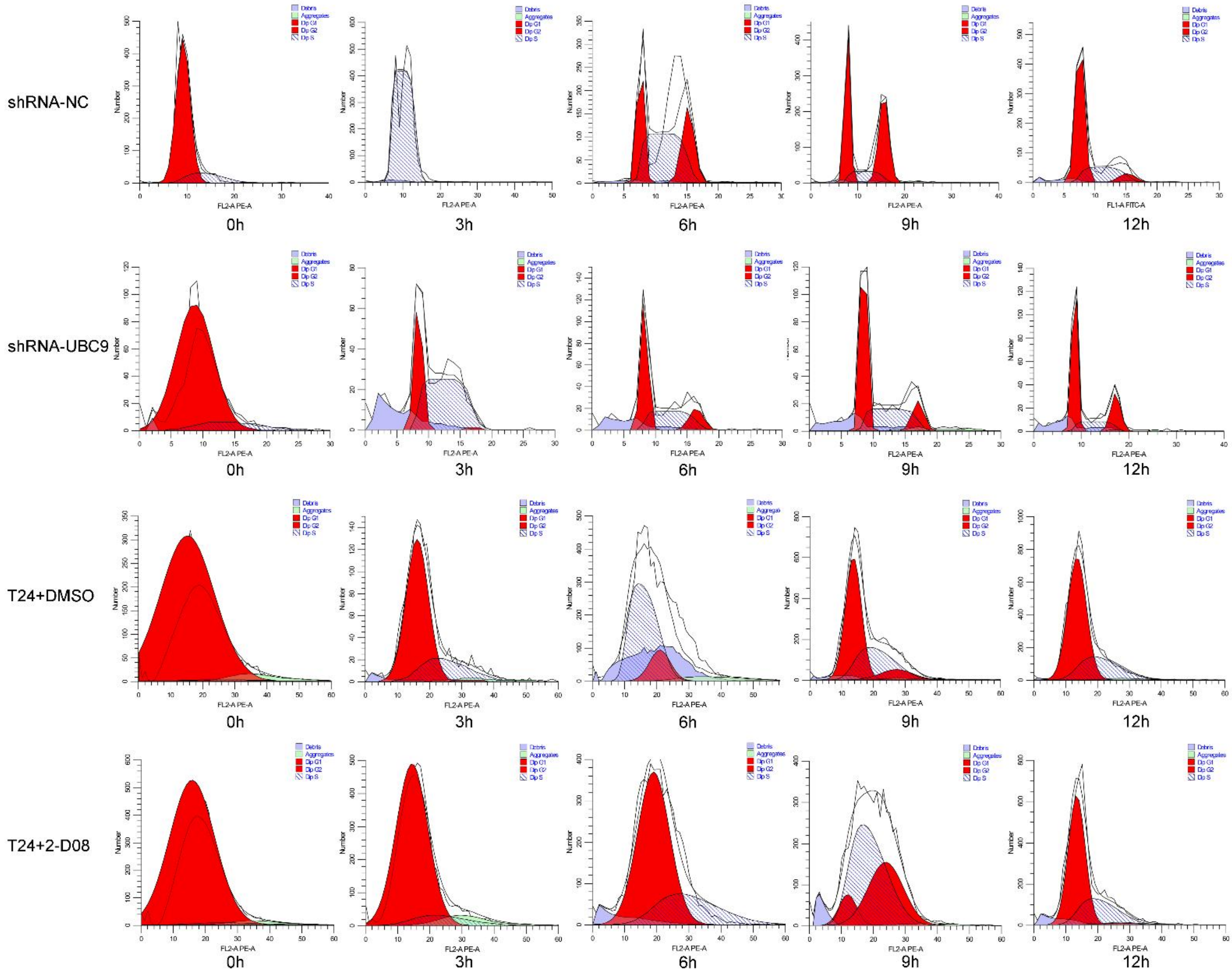

Figure S3: The flow charts of cell cycle synchronized using a double thymidine block. The vertical coordinates indicated cell counts and the horizontal coordinates indicated signal strength.

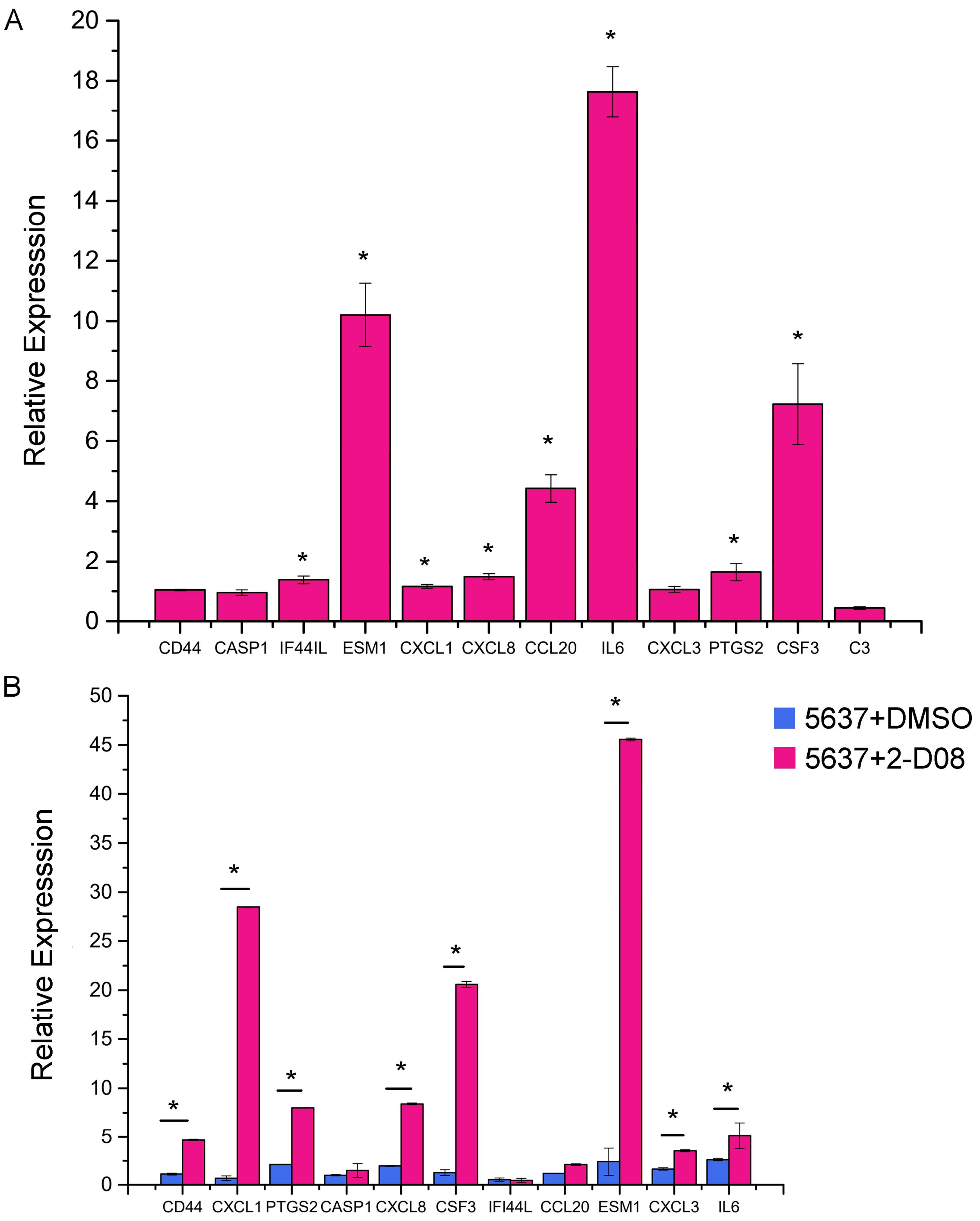

Figure S4: The expression of inflammatory factors after silencing of UBC9. (A) The expression levels of inflammatory factors after knock-down of UBC9; (B) The expression levels of inflammatory factors after 5637 cells treated with SUMOylation inhibitor 2-D08.

Table S1: The primers used in the study

Gene symbol	Primers	
UBC9	LEFT PRIMER	CAGGAAAGA AAGGGACTC
	RIGHT PRIMER	TTCGGGTGAAATAATGG
PTGS2	LEFT PRIMER	TAAGTGCGATTGTACCCGGAC
	RIGHT PRIMER	TTTGTAGCCATAGTCAGCATTGT
IL6	LEFT PRIMER	AGACAGCCACTCACCTCTTC
	RIGHT PRIMER	TTTCACCAGGCAAGTCTCCT
CXCL3	LEFT PRIMER	CGCCCAAACCGAAGTCATAG
	RIGHT PRIMER	GTCCTTTCCAGCTGTCCCTA
CCL20	LEFT PRIMER	CTCCTGGCTGCTTTGATGTC
	RIGHT PRIMER	ATTTGCGCACACAGACAACT
CXCL1	LEFT PRIMER	ACTCTACCTGCACACTGTCC
	RIGHT PRIMER	TCCCCTGCCTTCACAATGAT
IFI44L	LEFT PRIMER	GTTGAAAGATGCAGCCGTCA
	RIGHT PRIMER	AAACGACACACCAGTTGCTC
CXCL8	LEFT PRIMER	CAGTTTTGCCAAGGAGTGCT
	RIGHT PRIMER	ACTTCTCCACAACCCTCTGC
C3	LEFT PRIMER	TGTGCAGTCTCTAACCCAGG
	RIGHT PRIMER	TCCATTCGCAGGAGGAAGTT

Table S2: The association between UBC9 expression and clinical parameters in bladder cancer

clinical parameters	Case n	UBC9 staining n (%) Low expression	High expression	X2	P
Tissue				8.97	0.001**
Bladder cancer	106	16(15.1)	90(84.9)		
Adjacent normal tissues	14	8(57.1)	6(42.9)		
Sex				0.146	0.702
Male	93	15(16.1)	78(83.9)		
Female	13	1(7.7)	12(92.3)		
Age(years)				2.60	0.107
≤ 60	46	4(8.7)	42(91.3)		
> 60	60	12(20.0)	48(80.0)		
Tumor size(cm)				0.174	0.676
≤5	67	10(14.9)	57(85.1)		
>5	33	6(18.2)	27(81.8)		
Multifocality				0.381	0.537
single	43	8(18.6)	35(81.4)		
multiple	57	8(14.0)	49(86.0)		
pT stage				0.893	0.345
pT1-pT2	75	10(13.3)	65(86.7)		
pT3-pT4	25	6(24.0)	19(76.0)		
Grade				1.897	0.168
I-II	32	2(6.3)	30(93.8)		
III	74	14(18.9)	60(81.1)		

pT： Pathologic T category, **P ≤ 0.01
